# Risk factors for norovirus infection in healthcare workers during nosocomial outbreaks: a cross-sectional study

**DOI:** 10.1186/s13756-021-00979-8

**Published:** 2021-07-22

**Authors:** Kjell Torén, Linus Schiöler, Nancy P. Nenonen, Charles Hannoun, Anette Roth, Lars-Magnus Andersson, Johan Westin, Tomas Bergström

**Affiliations:** 1 School of Public Health and Community Medicine, Institute of Medicine, University of Gothenburg, Box 414, 405 30 Gothenburg, Sweden; 2 Department of Occupational and Environmental Medicine, Sahlgrenska University Hospital, Gothenburg, Sweden; 3 Department of Infectious Diseases/Virology, Institute of Biomedicine, University of Gothenburg, Gothenburg, Sweden

**Keywords:** Risk factors, Norovirus infection, Nosocomial outbreaks, Healthcare workers, Vomit, Rectal swabs

## Abstract

**Background:**

Norovirus outbreaks cause severe medico-socio-economic problems affecting healthcare workers and patients. The aim of the study was to investigate prevalence of norovirus infection and risk factors for infection in healthcare workers during nosocomial outbreaks.

**Methods:**

A cross-sectional study of norovirus infections in healthcare workers was performed in seven outbreak wards in a large university hospital. Packs (swab for rectal sampling, and questionnaire) were posted to healthcare workers on notification of a ward outbreak. Rectal samples were examined with norovirus-specific real-time PCR. Replies from questionnaires were analysed using logistic regression models with norovirus genogroup (G)II positive findings as dependent variable. The results are expressed as odds ratios (OR) with 95% confidence intervals (CI). Sequencing and phylogenetic analyses (1040 nucleotides) were used to characterize norovirus strains from healthcare workers. Cluster analyses included norovirus GII.4 strains detected in ward patients during the ongoing outbreaks.

**Results:**

Of 308 packs issued to healthcare workers, 129 (42%) were returned. norovirus GII was detected in 26 healthcare workers (20.2%). Work in cohort care (OR 4.8, 95% CI 1.4–16.3), work in wards for patients with dementia (OR 13.2, 95% CI 1.01–170.7), and having diarrhoea, loose stools or other gastrointestinal symptoms the last week (OR 7.7, 95% CI 2.5–27.2) were associated with increased norovirus prevalence in healthcare workers. Sequencing revealed norovirus GII.4 in healthcare workers samples, and strains detected in healthcare workers and ward patients during a given ward outbreak showed ≥ 99% similarity.

**Conclusion:**

Norovirus positive findings in healthcare workers were strongly associated with symptomatic infection, close contact with sick patients, and dementia nursing.

**Supplementary Information:**

The online version contains supplementary material available at 10.1186/s13756-021-00979-8.

## Introduction

Noroviruses (NoV) are a major cause of non-bacterial acute gastroenteritis affecting all age groups [1]. Infections are characterized by sudden onset of uncontrollable projectile vomiting, diarrhea, nausea and muscle pain. High levels of NoV are detected in vomit and faeces [2–4]. Transmitted by the faecal-oral route, NoV are implicated in gastro-enteric outbreaks linked to direct or indirect contact with the soiled hands, vomit, faeces, or aerosols from an infected individual as occurs in many settings including hospitals and restaurants [4, 5]. Outbreaks may also be traced to ingestion of sewage-contaminated water, bivalves, or food such as frozen berries contaminated by infected pickers or polluted water [5–10]. The routes of transmission are numerous as NoV are small, non-enveloped, environmentally stable RNA viruses, resistant to alcohol treatment and a wide range of temperatures from freezing to 60 °C [11–13]. These viral properties facilitate NoV spread in community and semi-closed settings where both healthcare workers (HCWs) and patients are affected [14–16].

As is typical of RNA viruses, the NoV, particularly genotype (G) II.4, evolve rapidly through mutation and recombination events, with the periodic emergence of new antigenic variants implicated in global outbreaks of acute gastroenteritis [17–19]. This genomic and antigenic variation may account for the apparent low immunity in the population following NoV GII infection, where around 30% of exposed subjects develop symptoms of acute gastroenteritis [3]. As with human influenza virus infections, genomic and antigenic variation of NoV, the individual´s immune status, and environmental conditions such as relative humidity and temperature may play a role in the recurring seasonality of NoV GII infections observed in community and healthcare settings [20].

Once introduced into the hospital NoV are readily transmitted to HCWs and patients in direct contact with infected individuals where droplet spread and aerosols from vomit and faeces may be implicated, or indirectly through contact with vomites, or by airborne transmission [4, 21, 22]. Viral shedding precedes onset of gastro-enteric symptoms, and low infectious dose ensures rapid spread within the ward [14, 23]. The elderly, the very young, and the immunosuppressed are highly susceptible to NoV, and prolonged symptomatic viral excretion is common in these patients [24–28]. Countermeasures including cohort care, enhanced hand-washing and environmental cleaning, place a heavy workload and responsibility on HCWs. Consequently, HCWs, patients and administrators confront disruptive delays in medical treatment, ward closures, and severe socio-economic problems. However, NoV transmission in HCWs and patients during nosocomial outbreaks is poorly understood [16, 26].

The aim of this cross-sectional study was to analyse risk factors for NoV infection in HCWs during nosocomial outbreaks.

## Methods

### Study outline

The study was carried out at Sahlgrenska University Hospital during nosocomial outbreaks of NoV, January to April 2012. The molecular epidemiology of these nosocomial NoV outbreaks has been described previously [4]. HCWs attending patients in seven wards in three separate hospital buildings were included. Ward nomenclature and medical units are shown in Table 1. Vomiting was defined as two or more episodes of vomiting in a 24 h period or three of more loose stools in a 24-h period. It could also be defined as one or more episodes of both vomiting and diarrhea in a 24-h period [29]. An outbreak ward was defined as a ward in which two or more patients presented with suspect or laboratory confirmed symptoms of acute NoV gastroenteritis (vomiting and/or diarrhea), and that the infection was spread to other patients within the ward [4]. Infection Control Officers defined the outbreaks and provided the virus laboratory with ward outbreak reports throughout the study.

**Table 1:** Questionnaire response and rectal swab sampling in healthcare workers during nosocomial outbreaks of norovirus in seven hospital wards: compliance and real-time detection of norovirus genogroup II

Hospital ward				Return of questionnaires and rectal swabs among health care workers				
Buil- ding	Ward	Floor	Care unit	Packs issu-ed^b^	Packs returned (%)	Response Time^c^ Range (median)	NoV GII^a^ Positive (%)	NoV GII^a^ Ct^d^ range (median)
A	A1	5th	Haematology^e^	41	19 (46%)	6–33 (18)	0	“– “
A	A2	5th	Medicine^e^	30	15 (50%)	5–21 (8)	0	“–“
B	B1	7th	Geriatric medicine	58	22 (38%(	2–28 (6)	1 (4.5%)	37
B	B2	6th	Geriatric orthopedy	52	26 (50%)^f^	7–13 (7.5)	9 (35%)	25–35 (28)
B	B3	6th	Geriatric medicine	48	16 (33%)	9–20 (11.5)	1 (6.3%)	35
C	C1	6th	Dementia	51	14 (27%)	6–11 (7)	7 (50%)	23–31 (30)
C	C2	4th	Substance abuse and dementia	28	17 (61%)	6–14 (8)	8 (47%)	22–34 (30)
All				308	129 (42%)	2–33 (8)	26 (20%)	22–37 (30)

^a^NoV GII: Norovirus genogroup II detection in real-time RT-PCR assays for NoV GI, GII, adenovirus, astrovirus, rotavirus, and sapovirus

^b^Sample packs: Q and swab for rectal sampling

^c^Response time: days taken to return sample packs containing completed Q for statistical analyses, and RS for viral analysis

^d^Ct: cycle threshold value in NoV GII specific real-time RT-PCR assay, the lower the Ct value the higher the viral load

^e^Interconnected wards: shared dining-room and shower facilities

^f^Two HCWs returned incomplete packs, these workers were excluded from the study

The HCW in each outbreak wards were identified using the personnel files, and we identified all workers employed in the wards. Cleaners were not employed by the hospital and were therefore not included in the study. On the day of notification of a ward outbreak, individual rectal swab (RS) sampling packs were posted to all HCWs in the outbreak ward, regardless if they were symptomatic or not. Packs, issued by the virus laboratory, contained a questionnaire, flocked swab for rectal sampling, sterile 10 ml tube, instruction form on RS self-sampling, and an addressed, prepaid envelope for return to the laboratory by standard post. Instructions described insertion of the swab 2 cm into the rectum, with light rotation before withdrawal [30]. The RS was broken off and placed in the empty sterile tube; capped tubes were labeled with HCW´s name and sampling date. HCWs were asked to return completed questionnaire, RS, and signed instruction form within one week.

### Questionnaire

The questionnaire was constructed with the aim to cover broad aspects of potential riskfactors for spread of virus in hospital wards. A pilot version of the questionnaire was tested on ten HCWs and after slight modifications, the final version comprised items covering age, gender, work-related factors, family and children, tobacco use, and current symptoms among the respondents. The wording of thirty key items covering different work-related factors are displayed in Table 2. Questions 1–8 (not shown) comprised items about age, gender, ethnicity, living conditions and occupational title. The full questionnaire is shown in the Additional file 1. For the final analysis certain items were merged to constructed variables “Visible faeces or vomit at the ward”; “Worked in different wards”; “Assisted patients with toilet or showering”; and “Cleaning up faeces or vomit”.

**Table 2:** Items used in the questionnaire and prevalence of affirmative answers divided upon health care workers: with negative or positive norovirus findings in faeces

Items		NoV detection in HCWs faeces		
		Negative N = 103	Positive N = 26	*P*-value
8	Have you read the hygiene instructions?			
	Yes	87.4%	76.0%	> 0.10
	Partially	4.9%	12.0%	
	No	7.8%	12.0%	
9	Do you follow the instructions?			
	Yes	91.2%	84.0%	0.04
	Partially	5.9%	0.0%	
	No	2.9%	16.0%	
*During the last seven days have there been;*				
10	visible faeces in your ward?	69.1%	88.0%	0.08
11	visible vomit in your ward?	44.2%	73.7%	0.02
*During the last seven days have you;*				
12	worked in different wards?	6.8%	19.2%	0.06
13	worked extra shifts in other wards?	2.9%	8.0%	0.3
14	transported patients between wards?	12.6%	19.2%	0.4
15	worked in an overcrowded ward?	40.0%	38.5%	1.0
16	worked with cohort care of NoV patients?	26.7%	57.7%	0.005
17	worked with other care of NoV patients?	31.4%	62.5%	0.009
18	worked with patients with diarrhoea of unknown cause?	49.5%	56.0%	0.7
19	handled sheets or clothes soiled with faeces?	65.7%	80.8%	0.2
20	handled sheets or clothes soiled with vomit?	17.5%	52.0%	0.001
21	cleaned spilled faeces?	51.0%	72.0%	0.07
22	cleaned spilled vomit?	5.8%	26.9%	0.005
23	changed infected patients’ napkins?	30.7%	69.2%	0.001
24	handled faeces in other ways, sample collection?	21.4%	38.5%	0.08
25	helped patients with showering?	68.6%	76.9%	0.5
26	helped patients with toilet visits?	80.2%	96.0%	0.07
28	handled food for patients?	63.1%	76.0%	0.3
29	assisted patients or their relatives using the ward´s kitchen?	9.1%	0%	0.2
30	during the same day cleaned up faeces or vomit and also distributed food to patients?	40.8%	28.0%	0.3
31	during the same day cleaned up faeces or vomit and also distributed food to the staff?	11.7%	3.8%	> 0.10
32	cooked your own food in the ward?	58.3%	76.9%	> 0.10
33	eaten nuts, snacks or goodies in the ward?	58.8%	38.5%	0.08
36	been together with staff from other wards?	13.6%	0%	0.07
37	eaten at the hospital canteen?	22.3%	12.0%	> 0.10
38	had lunch outside the hospital?	21.4%	16.0%	> 0.10

## Statistical methods

Descriptive statistics are presented as percentages or mean values with standard deviations (SD). The material was also analysed with logistic regression models. The dependent variable was detection of positive NoV GII in RS, and a logistic regression model adjusting for age and sex was run for each variable. Finally, a logistic regression model comprising all selected independent variables was applied using backward selection with a thresold of *p* < 0.2 to obtain the final model. The results from the regression models are presented as odds ratios (OR) with 95% confidence interval. All analyses were performed using the SAS statistical package (version 9.3).

### Virology studies

#### Real-time RT-PCR (rRT-PCR)

Total nucleic acids (TNA) were extracted from RS and screened for detection of NoV GI, GII, Sapovirus, Adenovirus, Rotavirus, Astrovirus in validated rRT-PCR systems, described previously [8, 30]. These assays provided semi-quantitative estimates of viral load based on cycle threshold values (C_t_) registered for each sample, and each viral agent. C_t_ values ≤ 38 were recorded as positive, where C_t_ values vary inversely with viral load, the lower the Ct value the higher the viral load [7]. Strict precautions were followed at each stage of sample handling to avoid cross contamination [31].

#### NoV GII RT-PCR, nucleotide (nt) sequencing and phylogenetic analyses

HCW samples with high viral load were amplified in gel-based RT-PCR of the NoV partial N/S-capsid-coding region (1040 nt), prior to nt sequencing [7]. NoV GII genotype was determined on sequence and phylogenetic analyses were performed as described previously [4, 32]. Cluster studies were based on comparative sequence and phylogenetic analyses of the NoV strains detected in HCWs. These analyses included NoV GII.4 strains detected in ward patients during the same nosocomial study period, and described previously [4].

## Results

Of 308 sample packs issued to HCWs in seven wards, 129 (42%) were returned to the laboratory with signed, completed questionnaires and RS (Table 1). HCWs in six wards responded within one week, as requested (Table 1). However, HCWs in ward A1, showed delayed response, but after additional information to HCWs the response rate was increased. The wards A1 and A2 were general internal medicine, the wards B1, B2 and B3 were for geriatric patients and the the wards C1 and C2 were psychiatric care, in which elderly patients with dementia dominated, why the doors were locked. In all wards there were two to four patients in each room, with a few single rooms.

Univariate analysis of subjects with positive NoV or negative NoV in relation to questionnaire items are shown in Table 2. Positive NoV was significantly associated with contact or handling vomit (several items); work with cohort care of NoV patients; work with other care of NoV, but there was no association of positive NoV in relation to preparing, eating or handling food. Less than 90% of the staff had read the hygiene instructions and around 90% followed the instructions. Those with low compliance to hygiene instructions had a significantly increased prevalence of NoV detection.

In Table 3 are shown that univariate analysis of descriptive data for the participants. Having gastronintestinal symptoms and work in wards for patients with dementia was significantly associated with positive NoV. Other factors like smoking habits, having children at day care centre or number of cohabitants in household were not related to positive NoV.

**Table 3:** Descriptive data of the study subjects

Variable	All	Negative NoV^a^	Positive NoV^a^	*P*-value
All	129 (100.0%)	103 (79.8%)	26 (20.2%)	
Age, mean (SD)	47.2 (12.1)	46.7 (11.6)	49.2 (11.3)	
Women	112 (86.8%)	91 (88.3%)	21 (80.8%)	0.3
Current smokers	16 (12.4%)	13 (12.6%)	3 (11.5%)	1.0
Living as single	25 (19.4%)	17 (16.5%)	8 (30.8%)	0.2
Mean number (SD) of cohabitants in household	1.7 (1.3)	1.7 (1.3)	1.2 (1.1)	0.2
Children < 18 yr	38 (30.9%)	35 (35.0%)	3 (13.0%)	0.05
Children at daycare centre	14 (11.5%)	12 (12.1%)	2 (8.7%)	1.0
Worked as nurse	110 (85.3%)	85 (82.5%)	25 (96.2%)	0.1
Worked in wards for patients with dementia	57 (44.2%)	33 (32.0%)	24 (92.3%)	< 0.0001
Have you had diarrhea or loose stools the last 7 days?	40 (31.3%)	22 (21.6%)	18 (69.2%)	< 0.0001
Have you had other GI^b^ complaints the last 7 days?	20 (15.9%)	11 (10.9%)	9 (36.0%)	0.005
Have you vomited the last 7 days?	12 (9.4%)	2 (2.0%)	10 (38.5%)	0.0001
Have you had a cold the last 7 days?	39 (30.5%)	34 (33.3%)	5 (19.2%)	0.2

^a^NoV = Norovirus real-time RT-PCR findings detected in HCWs faeces

^b^GI = gastrointestinal

In Table 4 are shown the logistic regression models for separte included variables adjusted for age and gender. Increased prevalence of positive NoV detection was observed in relation to “Cleaned up faeces or vomit” (OR 5.0, 95% CI 1.9–14.7), “worked in cohort care” (OR 4.6, 95% CI 1.7–13.2), and “worked in wards with dementia patients” (OR 12.8, 95% CI 1.5–106.3).

**Table 4:** Logistic regression models adjusting for age and gender using norovirus positive findings in health care worker faeces as dependent variable

Independent variable	Odds ratio, OR	95% confidence interval	*P*-value
Visible faeces or vomit at the ward	2.90	0.90–12.53	0.11
Worked in different wards	1.67	0.55–4.73	0.35
Worked in an overcrowded ward	1.22	0.46–3.19	0.69
Worked with cohort care	4.6	1.71–13.24	0.004
Worked with other care (not cohort)	3.4	1.31–9.56	0.015
Handled faeces or vomit	5.0	1.90–14.74	0.002
Assisted patients with toilet or showering	4.1	0.81–48.18	0.16
Current smoking	1.6	0.34–6.18	0.54
Diarrhea, loose stools or other gastrointestinal symptoms	15.1	4.86–59.94	0.0001
Worked in wards for patients with dementia	12.8	1.53–106.31	0.005
Worked as nurse	1.9	0.32–11.13	0.29

In the final logistic regression model, adjusting for gender and age, and with backward selection for all selected variables, resulted in an association between positive NoV and “worked with cohort care” (OR 4.8, 95% CI 1.4–16.3), “worked in wards for patients with dementia” (OR 13.2, 95% CI 1.01–170.7), and having diarrhoea, loose stools or other gastrointestinal symptoms the last week (OR 7.7, 95% CI 2.5–27.2).

### Virology studies

#### Real-time RT-PCR analysis

NoV GII was detected in 26 of the 129 compliant HCWs (20%) on rRT-PCR analysis of TNA extracts prepared from RS. These samples showed NoV C_t_ range 22–37, median 30 (Table 1); no other enteric viral agents were detected.

#### NoV GII RT-PCR, nucleotide sequencing and phylogenetic analyses

Sequence-based genotyping of NoV GII rRT-PCR positive samples revealed NoV GII.4 strains in RS from twenty HCWs working in three outbreak wards (B2, C1, and C2) within buildings B and C (Table 1). These GII.4 strains, detected in HCW samples from the nosocomial outbreaks (Gothenburg 2012), sequenced as NoV GII.4 strains most closely related to NoV GII.4 sub-type NewOrleans 2009.

**Fig. 1:**
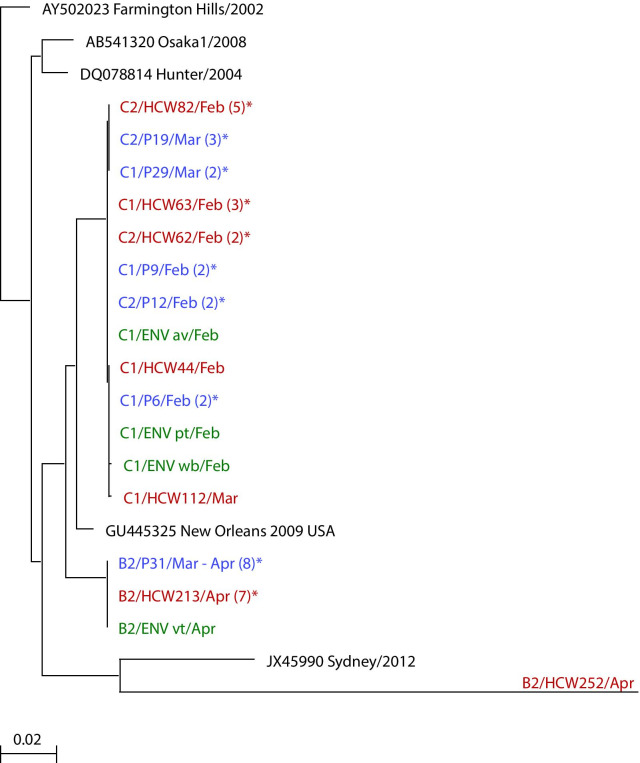
Phylogenetic analysis of NoV GII sequences (1 kb) detected in 20 healthcare workers (HCW) from outbreak wards B2, C1 and C2 (Table 1), spring 2012. Representative NoV GII.4 sequences from HCWs in ward B2, C1or C2 are shown in red as HCWidn (HCW identification number); The non-GII.4 strain, from HCW252, is included. Pidn (blue) represents GII.4 sequences reported previously from patients in the given ward. The number of identical sequences from the ward are outlined as (n)*. ENV (green) indicates NoV GII.4 sequences from environmental sites in the given ward: pt (patient table), wb (wash-hand basin), av (air vent), vt (virus trap)^2^ Relevant NoV GII.4 reference strains, with GenBank accession numbers, are included. The bar indicates genetic distance per nucleotide/site

Comparative sequence analyses of the strains detected in HCWs revealed ≥ 99% nucleotide similarity (1040 nt) between NoV GII.4 strains from HCWs working in a given ward, at a given time, and the GII.4 strains reported from patients in that ward at that time [4]. Phylogenetic analyses illustrated clustering of NoV GII.4 strains detected in HCWs and patients from a given outbreak ward (Fig. 1). Sine NoV strains from these wards clustered on separate branches, thay can be regarded as distinct outbreaks. Notably, as an exception, strains detected in HCWs and patients from wards C1 and C2, situated on different floors in building C, placed on the same sub-branch throughout the study period (Fig. 1, Table 1) [4].

## Discussion

The most interesting result from this cross-sectional study of HCWs was that person-to-person contact was strongly associated with positive NoV findings in faeces, while there was no association with food handling or working in different wards. Further, work in wards caring for highly dependent patients, i.e. patients with dementia, was strongly associated with positive norovirus findings among the personell. Work in psychiatric wards or in nursing homes has been associated with norovirus outbreaks, especially in large facilities [33]. Work with cohort care was also strongly associated with positive NoV findings, despite hygiene measures taken in these facilities. Increased airborne levels of norovirus have also been measured close to vomiting patients with norovirus infections [34]. Moreover, positive NoV GII findings in HCWs were also clearly associated with acute gastrointestinal symptoms.

Representative NoV strains from HCW samples sequenced as NoV GII.4, a genotype commonly detected in patients during hospital outbreaks [4, 35, 36]. Comparative sequence and phylogenetic analyses indicated that the NoV strains detected in HCWs and in patients during nosocomial outbreaks in Gothenburg 2012 were most closely related to NoV GII.4 subtype NewOrleans2009. Furthermore, the NoV strains detected in HCWs and in patients from a given ward at a given time showed ≥ 99% similarity (1040 nt) [4]. This finding indicates ongoing NoV cross-infection between patients and staff occuring in each ward, during the outbreak setting.

Nursing staff in wards caring for long-term, incapacitated, dement, or substance abuse patients whose mobility was difficult to restrict, were most affected by the nosocomial outbreaks (Table 1) [4]. These findings agree with previous reports on NoV infections in HCVs working in psychiatric and long-term care units where close patient-staff contact, environmental contamination, and difficulties in confining index patients to their rooms, contributed to prolonged outbreaks [34, 37].

Notably, psychiatric aides in the dementia wards responded rapidly to requests for prompt return of sample packs, indicating their concern to improve the immediate pressure of work situation within the long-term care units, where environmental contamination was also evident [4]. Moreover, NoV strains detected in HCWs in wards C1 and C2, situated in the same building two floors apart (Table 1), were highly similar (Fig. 1). This suggests that psychiatric aides were sharing heir work between these two wards, a difficult work situation (Table 1).

In contrast, despite assurances of anonymity, poor or delayed response was noted in HCWs from ward A1, where participants were highly-trained nursing staff caring for short-term patients (Table 1). This delay may have had a deleterious effect on the outcome of molecular studies (NoV detection and sequencing) as indicated by the negative results from HCWs in wards A1 and A2. The problems of delayed sampling when assessing detection and clearance of NoV in healthy HCWs are recognized [38]. In a previous report, 13% of HCWs described diarrhoeal symptoms in the absence of faecal NoV, findings which the authors associated with delayed sampling [38]. Similarly, 21% of HCWs in the present study described diarrhoeal symptoms but NoV was not detected in their RS samples.

Low compliance and the slow response in providing samples proved major limitations in the study of NoV infections in HCWs. Against this background of low compliance, no on-going or follow-up HCW sampling was undertaken/attempted.

This cross-sectional study of HCWs carried out during nosocomial outbreaks in hospital wards showed that NoV infections were strongly associated with symptomatic infection, close contact with sick patients, and dementia nursing.

Since work with cohort care was also strongly associated with positive NoV findings, hygiene measures need to be further improved to control viral spread to HCW during nosocomial outbreaks. Hence, several additional recommendations were presented to the Department of the Infection Control;

Staff were recommended not to work at more that one ward during outbreak. Current hygiene routines regarding the staff handling of faeces and vomits were evidently not sufficiently strict, and improved hygiene in form of obligatory hand washing after removal of gloves was suggested.

However, the also indicated that additional studies focusing on possible environmental /aerosol spread of NoV between HCW and patients also seems warranted [4, 22, 34].

## Supplementary Information

**Additional file 1.** The complete questionnaire.

## Data Availability

The data can be available upon reasonable request to the corresponding authors after and approval from the Swedish Committee of Ethics**.**

## Abbreviations

OR: Odds ratio
CI: Confidence interval
NoV: Noroviruses
rRT-PCR: Real-time RT-PCR
TNA: Total nucleic acids
G: Genotype
HCWs: Healthcare workers
C_t_: Cycle threshold values
nt: Nucleotide
