# Supplementary material for: Risk factors for norovirus infection in healthcare workers during nosocomial outbreaks: a cross-sectional study
Source: Antimicrob Resist Infect Control. 2021 Jul 22;10:107. doi: 10.1186/s13756-021-00979-8 (PMC8299649; doi:10.1186/s13756-021-00979-8)
Supplement: Supplementary file 1 — Additional file 1. The complete questionnaire. [file 13756_2021_979_MOESM1_ESM.pdf]

We have initiated a research project to study risk factors for Calici gastroenteritis (NoV disease). Hence, we want to put a number of questions to you.

Name________________________________ **Date**_______________________________

Cellular________________________________ **Phone** ______________________________

FACTS ABOUT YOURSELF

1. How old are you? _______ years
2. Are you a man or a woman? ❒ Woman ❒ Man
3. In which country are you born? ❒ Sweden ❒ Another country, which __________
4. Which is your current civil status? ❒ Married ❒ Single
5. How many persons do you live with? _________

OUESTIONS ABOUT YOUR WORK

6. Which is your current occupation (nurse, auxiliary nurse etc.)?_______________________
7. Are there written hygiene instructions in your work? ❒ Yes ❒ No
8. Have you read the hygiene instructions? ❒ Yes ❒ Partially ❒ No
9. Do you follow the instructions? ❒ Yes ❒ Partially ❒ No
10. During the last seven days, have there been visible faeces in your ward? ❒ Yes ❒ No
11. During the last seven days, have there been visible vomit in your ward? ❒ Yes ❒ No
12. Have you the last seven days worked in different wards? ❒ Yes ❒ No
13. Have you the last seven days worked extra shifts in other wards? ❒ Yes ❒ No

14. Have you the last seven days transported patients between wards?

❐ Yes ❐ No

15. Have you the last seven days worked in an overcrowded ward?

❐ Yes ❐ No

16 Have you the last seven days worked with cohort care of NoV patients ?

❐ Yes ❐ No

17 Have you the last seven days worked with patients with known NoV (not cohort care)?

❐ Yes ❐ No

18 Have you the last seven days worked with patients with diarrhoea of unknown cause?

❐ Yes ❐ No

19 Have you the last seven days handled sheets or clothes soiled with faeces?

❐ Yes ❐ No

20 Have you the last seven days handled sheets or clothes soiled with vomit?

❐ Yes ❐ No

21 Have you the last seven days cleaned spilled faeces? ❐ Yes ❐ No

22 Have you the last seven days cleaned spilled vomit? ❐ Yes ❐ No

23 Have you the last seven days changed NoV infected patients' napkins?

❐ Yes ❐ No

24. Have you the last seven days handled feces in other ways, such as sample collections?

❐ Yes ❐ No

25 Have you the last seven days helped patients with showering? ❐ Yes ❐ No

26 Have you the last seven days helped patients with toilet visits? ❐ Ja ❐ Nej

27 How many patients, approximately, have you helped with showering or toilet visits the last seven days? ________________

28 Have you the last seven days handled food for patients? ❐ Yes ❐ No

29 Have you during the last seven days assisted patients or their relatives using the ward's kitchen? ❐ Yes ❐ No

30 Have you the last seven days during the same day cleaned faeces or vomit, and also distributed food to patients? ❐ Yes ❐ No

31 Have you the last seven days during the same day cleaned faeces or vomit, and also distributed food to the staff? ❐ Yes ❐ No

32. Have you the last seven days cooked your own food in the ward? ❐ Yes ❐ No

33. Have you the last seven days eaten nuts, snacks, or goodies in the ward? ❐ Yes ❐ No

34. Have you the last seven days had coffee/tea with the staff;

34.1 ❐ at the ward?

34.2 ❐ outside the ward?

35. Have you the last seven days had coffee/tea with patients? ❐ Yes ❐ No

36. Have you the last seven days had coffee/tea with staff from other wards? ❐ Yes ❐ No

37. Have you the last seven days had lunch at the hospital canteen? ❐ Yes ❐ No

38. Have you the last seven days had lunch outside the hospital? ❐ Yes ❐ No

39. Have you the last seven days used the patient's toilet? ❐ Yes ❐ No

QUESTIONS ABOUT YOUR FAMILY

40. Have you children younger than 18 years living at home? ❐ Yes ❐ No
If "No", continue to item 47.
If "Yes"; go to next item.

41.1 How many children do you have? ______________

41.2 How old are they? ______________

42. Are anyone of the children attending day-care? ❐ Yes ❐ No

43. Do you have a childminder (au-pair) to any of the children? ❐ Yes ❐ No

44. Have any of the children had diarrhoea or loose stools the last seven days? ❐ Yes ❐ No

45. Have any of the children had fever the last seven days? ❐ Yes ❐ No

46. Has there been NoV disease at your children's day-care center or school the last seven days? ❐ Yes ❐ No

47. Has anyone in your household had diarrhoea/loose stools the last seven days? ❐ Yes ❐ No

48. Has there been NoV disease in any of your family's workplace the last seven days?
❐ Yes ❐ No

SMOKING HABITS

49. Have you ever smoked daily during at least one month? ❐ Yes ❐ No
If "No", continue to item 52.
If "Yes", go to next item.

50. Are you still smoking? ❐ Yes ❐ No

51. Have you smoked at the ward the last seven days? ❐ Yes ❐ No

52. Have you snuffed daily during at least one year? ❐ Yes ❐ No

53. Are you still snuffing? ❐ Yes ❐ No

54. Have you snuffed the last seven days when you were at the ward? ❐ Yes ❐ No

QUESTIONS ABOUT YOUR HEALTH

Have you had or have;

55. Psoriasis? ❐ Yes ❐ No
56. Diabetes? ❐ Yes ❐ No
57. Inflammatory gut disease? ❐ Yes ❐ No
58. Disease with immunosuppression? ❐ Yes ❐ No
59. Hayfever? ❐ Yes ❐ No

60. Have you ever had nasal symptoms like stuffy nose, rhinorea, or sneezing without having a cold? ❐ Yes ❐ No

61 Have you had diarrhoea/loose stools the last seven days? ❐ Yes ❐ No

62 Have you had any other intestinal symptoms the last seven days? ❐ Yes ❐ No

63 Have you vomited at any time the last seven days? ❐ Yes ❐ No

64 Have you had a cold or fever during the last seven days? ❐ Yes ❐ No
